# On the Use and Administration of Cod-Liver Oil in Pulmonary Consumption

**Published:** 1850-01

**Authors:** C. J. B. Williams

**Affiliations:** Prof. of Medicine in University College, London; Consulting Physician to the Hospital for Consumption, etc.


### ARTICLE II.

*On the Use and Administration of Cod-Liver Oil in Pulmonary Consumption.*—By C. J. B. Williams, M.D., F.R.S., Prof. of Medicine in University College, London; Consulting Physician to the Hospital for Consumption, etc. (From the London Journal of Medicine.)

There is no department of medical knowledge, which seems to me to stand so much in need of improvement, as that which relates to the operation of medicines. Even with regard to those most commonly used, it is surprising what a diversity of opinion prevails among different practitioners; and, as a necessary consequence, there is almost equal variation in the modes and combinations in which each medicine is administered. Yet, it is pretty obvious, that as truth is essentially simple and constant, that there must be much of error in such diversity of opinion and practice, and the sooner the the truth is elicited by a careful and rational examination of facts bearing upon each subject, the more safe and satisfactory will our practice become.

The remedial influence of the Cod-liver oil particularly deserves this kind of investigation; not only because its mode of operation is a subject of much difference of opinion, but because the effects ascribed to it by many practitioners are of a very palpable and positive kind; and because such effects have not hitherto been obtained from other remedial agents.—The object of the present communication is to record the chief results of my own experience in the use of this remedy, in tuberculous and analogous diseases of the lungs. These results will be arranged briefly under the following heads:

1. General results of the use of Cod-liver oil in Phthisis Pulmonalis.
2. On its mode of operation.
3. On its preparation and administration.

### 1. *General Results of the Use of Cod-Liver Oil in Pulmonary Consumption.*

I have prescribed the oil in above four hundred cases of tuberculous disease of the lungs in different stages, which have been under my care in private practice, during the last two years and a half. Of these, I have 234 cases recorded in my note-books, with the results of the treatment at various intervals; these constitute the chief materials of the present communication.

Out of this number, the oil disagreed, and was discontinued, in only nine instances. In nineteen, although taken, it appeared to do no good; whilst in the large proportion of 206 out of 234, its use was followed by marked and unequivocal improvement; this improvement varying in degree in different cases, from a temporary retardation of the progress of the disease, and a mitigation of distressing symptoms, up to a more or less complete restoration to apparent health.

The most numerous examples of decided and lasting improvement, amounting to nearly 100, occurred in patients in what is usually termed the second stage of the disease, in which tuberculous deposits begin to undergo the process of softening, the common physical signs being defective movement and breath-sound, with muco-crepitation and marked dulness below or above a clavicle, or above a scapula, and tubular breath and voice-sounds towards the root, or inner part of the apex of tho same lung. Such patients generally have had cough for some months, latterly with muco-purulent or opaque yellowish or greenish expectoration, and have begun to lose flesh, color, and breath, in such a degree as to excite alarm, and induce them to seek further advice. With many, night-sweats had occasionally occurred; and hæmoptysis may have been present at a former period.

The effect of the Cod-liver oil in most of these cases was very remarkable. Even in a few days, the cough was mitigated, the expectoration diminished in quantity and opacity; the night-sweats ceased; the pulse became slower and of better volume; and the appetite, flesh, and strength were gradually improved. The first change manifest in the physical signs was generally a diminution and gradual cessation of the crepitus; the breath-sound becoming drier and clearer; but the dullnes, and tubular character of the breath and voice-sounds were much more persistent, and rarely exhibited a marked decrease, until after several weeks' use of this remedy, in conjunction with regular counter-irritation. The tubular sounds, in fact, frequently became louder at the first removal of the crepitus, which in phthisis as well as in pneumonia, tends to mask the signs of consolidation. In several instances, however, in which I have had the opportunity of examining the patients under treatment, at several successive intervals of a month or six weeks, the gradual removal of the consolidations has been unequivocally proved, by the restoration of clearer vesicular breath and stroke-sounds tothe affected spots. In several cases, in which disease has existed long, the restoration has never been perfect; even where the health has been completely re-established, and all common symptoms of disease have entirely disappeared, there have remained perceptible inequalities in the breath and stroke-sounds; generally, with prolonged expiratory sound, which has more or less of a tubular note towards the root of the lung of the same side. These signs, if unaccompanied by decided dulness on purcussion, I have learnt by the experience of many years, not to consider as exceptional against recovery, for they appear to be dependent on the puckering of the texture, often with pleural adhesions and old deposits in the bronchial glands, so frequently found after death at the summits and near the roots of the lungs of persons who have not for many years exhibited symptoms of any pectoral disease.

As might be anticipated, a large number of the phthisic patients for whom I have been consulted, have been in the first stage of the disease, in which the tubercles or deposits are in the solid state. In these cases, also, I have largely used the Cod-liver oil, and, so far as I have ascertained them, with not less satisfactory results; but a large proportion of these patients I have been unable to add to the numbers mentioned above, from my having seen them only once, or not frequently enough to enable me to determine with accuracy the results of the treatment. Such patients do not commonly consider themselves sufficiently il to be under constant medical treatment; and although the good effect of the oil is commonly manifest in the abatement of cough and feverish excitement, and in the improvement of flesh and strength, yet the benefit is less speedy and obvious than in more advanced stages of the malady. The physical signs of improvement are precisely the same as those which take place tardily in the second stage after the removal of the humid rhonchi; and in truth, the treatment by the oil combined with counter-irritation, where successful, seems to bring back the lungs from the second stage, that of incipient softening, to the first stage, that of simple deposit, whicn is tardier in its changes of increase or diminution, and may remain long stationary without any obvious alteration. The remark is applicable to the chronic products of inflammation of the lung, which, as is known to the profession, I consider to aproximate in nature to the higher class of tuberculous deposits.

The most striking instance of the beneficial operation of Cod-liver oil in phthisis, is to be found in cases in the third stage,—even those far advanced, where consumption has not only excavated the lungs, but is rapidly wasting the whole body, with copious purulent expectoration, hectic, night-sweats, colliquative diarrhœa, and other elements of that destructive process by which, in a few weeks, the finest and fairest of the human family may be sunk to the grave.

The whole number of cases in the third stage of phthisis, (that is, with one or more cavities, as indicated by physical signs) which have been manifestly improved under treatment with Cod-liver oil, amounts to sixty-two, up to the end of August. In thirty-four of these, I know that the improvement has continued up to a recent period, when I saw the patients, or had reports. Eleven cases, which exhibited decided improvement for a time have since again declined or terminated in death. Of the remaining seventeen I have had no recent report, and I do not know whether the amelioration has been permanent or not.

The results above stated give to Cod-liver oil, even as a tardative or palliative in phthisis, a rank far above any agent hitherto recommended, whether medicinal or regiminal. I have made extensive trials of several other medicines of reputed utility in the disease, and on a future occasion may lay before the profession the results of my experience, which prove some of these agents to be by no means inoperative or useless; and I still consider them to be often salutary aids in the treatment of this formidable malady, but their utility and harmlessnes fall so far short of those of the Cod-liver oil, that

I regard them now chiefly as subsidiary means, and the more likely to be useful, in proportion as they facilitate the exhibition or continuance of this superior ageut.

If the experience of the profession at large should accord with my own, and that those of who have preceded me in recommending the Cod-liver oil, our prognosis with regard to phthisis must undergo some modification. To what extent this modification may reach, cannot be determined, until such cases as those which I have recorded have been tested by years of time, but even now, when we repeatedly find forms and degrees of disease, that former experience had taught us to be utterly hopeless and speedily fatal, retarded, arrested, nay sometimes even removed and almost obliterated by various processes of restored health, we must pause ere we, in future, pass the terrible sentence of "no hope" on the consumptive invalid.

### 2. *Mode of Operation of Cod-Liver Oil.*

It seems scarcely necessary to discuss the question, whether oil owes its efficacy to the iodine which it contains. The amount of this element is so minute as hardly to admit of quantitive measurement; and to ascribe virtue to such infinitesimal fractions, when ordinary doses have no corresponding activity, is to adopt the fanciful and mischievous speculations of the homœopathists, which cannot be too strongly deprecated by the scientific and conscientious practitioner. Several of the patients whose cases are cited above, and many more of whom I have records, had taken iodine in various combinations before taking the oil, but without any effects approaching to those which ensued on the change of treatment. I am by no means incredulous of the salutary operation of iodine in some forms of tuberculous and scrofulous disease; inded, until I used the pure oil, I considered it to be the most useful remedy; but in the last two years, the oil has so far surpassed it and every other medicine in beneficial operation, that I am convinced that it acts by a virtue peculiar to itself.

The Cod-liver oil is a highly nutrient material; and it is commonly admitted by all practitioners who have used it, that it possesses, in a pre-eminent degree, the property of fattening those who take it for any length of time. But its nourishing influence extends beyond the mere deposition of fat in the adipose tissue. The muscular strength and activity are sensibly and sometimes rapidly increased under its use; whilst the improved color of the cheek and lips implies a filling of the vessels with more and better blood. Researches are wanted, to elucidate this subject more clearly; but the analysis of the blood in one case of phthisis which had been under treatment by the oil, showed a most remarkable increase of the animal principles of the blood, especially the albumen, which amounted to thirteen per cent., being nearly double its usual amount, whilst the fat was not materially augmented; and the fibrin, which is generally high in phthisis, was reduced below the normal proportion. If these results should be confirmed by further observation, there will be no difficulty in understanding that the Cod-liver oil should prove a nutrient to all the textures; although it may yet be a question, whether it does so by direct conversion into albumen or fibrin, or by preventing the waste of the albuminous principle by protecting it from the action of the oxygen absorbed in respiration.

But there is much reason to believe that the oil itself proves serviceable in supplying the fat molecules which appear to be essential to healthy nutrition, as forming the nucleoli of the primary cells or rudiments of tissues. The important part which fat thus performs in the process of nutrition, was first pointed out by Ascherson of Berlin; and that fat forms the central molecules of the elementary granules and cytoblasts of textures, is generally admitted, although few agree with Ascherson in his opinion that the fat forms the cells by its power of coagulating albumen around it. It seems to have been the opinion of Dr. Ascherson and of Dr. Hughes Bennett, who cites it, that in scrofulous diseases there is a want of this fat, and that the albumen derived from the food in digestion is liable to be precipitated in an unorganizable condition (as tubercle, etc.) for the lack of it. But it is now well ascertained that scrofulous and tuberculous deposits, so far from being deficient in fatty particles, contain them in greater quantity than exists in the blood, or in its plasma in a healthy state. The explanation which I have given of the chief salutary action of the cod-liver oil, is not that it supplies fat where it is wanting, but that it supplies fat of a better kind, more fluid, more divisible, less prone to change, and more capable of being absorbed into, and of pervading, the structures of the body: thus affording a fine "molecular base" in the chyle, and therein, a material for a better plasma; and being conveyed into the blood distributed through capillaries and around deposits (in such quantity as to soften and dissolve the crystaline and irregularly concreted fat scattered through them,) it renders them more amenable to the process of reparation and absorption. Hence its beneficial operation is more marked in those stages of tuberculous disease in which the deposits abound in fat: that is, at the period of maturation and softening; although from the extent of mischief already done both to the part and to the system, the benefit may not be so lasting as in the early stages of the disease.

One of the most remarkable effects of the cod-liver oil, in some cases of the second and third stage of phthisis, and in other forms of scrofulous disease with extensive suppuration, is the speedy removal of the sweats and other symptoms of hectic fever. This can hardly be ascribed to its direct nutrient powers; but I think that it is due to its influence in diminishing the unhealthy suppuration which is excited around the softening and excavated tubercles. If my views of the chemical nature of suppuration,—that it consists of a further oxydation of the exudation corpuscle,—be correct, then it is quite intelligible that the presence of so highly combustible a material as oil must check this process of oxydation, and thus prevent the degeneration of the corpuscles into the aplastic state of pus globules. In fact, if it should prove to be correct, according to the analysis above quoted from Simon, that cod-liver oil removes the excess of fibrin in the blood of phthisical patients,—this also equally accords with my notion, founded on the inferences of Mulder and others, that the formation of fibrine is due to a process of oxydation of the albumen (forming a deutoxide of protein, according to Mulder;) and that, by preventing this, the oil removes that tendency to cacoplastic inflammatory deposits which largely contribute to increase the consolidation of the lungs and other organs in phthisical subjects.

In making these surmises, I would not be supposed to adopt the idea of Liebig, that pulmonary consumption is the result of an excess of oxygen in the blood at large, consuming its materials, and those of the textures. Many of the symptoms, as well as the organic lesions of the disease, show that there is a great deficiency in the process of respiratiou by which oxygen is supplied to the blood; and some of the most rapidly fatal cases, exhibiting speedy emaciation, are, throughout their course, in a condition bordering on asphyxia. Here is obviously a great want of oxygen in the blood,—nay, I believe the excess of fat in the liver, and in the tuberculous deposits, in these instances, to be caused by this very scanty supply of oxygen to the system. But although it is deficient in the system, enough oxygen comes into contact with the exudations from cavities in the lungs, and from the diseased bronchi in their vicinity, to effect the formation of much unhealthy pus; and it is the formation and reabsorption of this that seems to excite the hectic of phthisis, as well as as to keep up much harassing local irritation. Now, I believe it to be by diminishing these exudations, and checking their further oxydation into pus, that cod-liver oil acts so promptly in reducing the hectic sweats and purulent expectoration of phthisis, which accelerate and aggravate its destructive progress.

The limits of this paper will allow me to notice but briefly one more point in regard to the action of cod-liver oil. Unlike other oils or fats, it rarely disorders the stomach or bowels, or disturbs the functions of the liver. If taken in any quantity, vegetable oils commonly purge, animal oils turn rancid in the stomach, causing heartburn, bilious attacks, and even jaundice. On the contrary, cod-liver oil generally improves all the chylopoietic functions, and distinctly promotes the action of the liver; so that the appetite and power of digestion are restored, and patients are enabled to take an amount and variety of food beyond what they were accustomed to, even in health. I cannot help thinking, that this peptic influence of the oil is due to its containing some biliary principle, which both favors its divisibility in the process of digestion, and promotes the natural secretions of the liver. The flow of bile, as indicated by the color of the fæces, is generally free and uniform during its exhibition; and I must not omit to notice another fact, which I believe to be connected with increased activity of the liver. I have in numerons instances remarked that the bulk of the liver (as determined by percussion) becomes augmented during its use; yet without tenderness or any other sign of disorder. In fact, this seems to be a kind of useful hypertrophy, induced by the oil augmenting the bulk and quantity of the hepatic cells, and supplying at once a material the more fitted for this secretion, because it has already within it some elements of biliary matter which served a similar purpose in the liver of the fish, and this at a lower temperature, and less favorable to the activity of the process. The observation of this influence of cod-liver oil has led me to use it in several cases of functional and structural disease of the liver, marked by defective or depraved secretion, and in some instances with most satisfactory results, especially in one of habitual formation of gall-stones, which had resisted all kinds of treatment, and was rapidly destroying the health: the use of the oil has entirely stopped the attacks, and has restored the patient to good health.

It appears probable, therefore, that although other oils might be equally influential in promoting nutrition, and in preventing and removing the cacoplastic and aplastic exudations of scrofulous subjects, the oil from the cod's liver, and perhaps those from the livers of other fish, have the advantage in point of digestibility, and in promoting the action of the digestive and biliary organs.

### 3. *Preparation and Administration of Cod-Liver Oil.*

It may seem somewhat strange that this remedy, which has been long employed and valued on the continent, and in some limited localities in this country, and of late years has been strongly urged on the attention of practitioners, both at home and abroad should have been so slow in being received into general use. If the experience of other practitioners accords with my own on this point, I would give as the reason of this tardy introduction, the disgusting smell and taste of the oil as it has been commonly prepared, and an impression generally pevalent that the efficacy of the remedy is connected with these offensive properties. This notion was favored by Dr. Hughes Bennett, in his monograph published in 1841. At that time I made several trials of the oils, selecting the clearer specimens of the brown oil, as recommended; but I found that so few patients could take it at all, and fewer still were able to persevere with it, that the inference seemed to be, that however German and Dutch stomachs might bear it, English ones could not, at least among the upper classes. It was not until I had witnessed some striking examples of benefit ensuing from the use of the pure oil, prepared according to Mr. Donovan's method, that I began again to make trial of it, and to reflect further on its mode of operation when freed from all impurities. The value of the oil will be much increased by the statement that in all instances I have prescribed it as *free from taste and smell as could be procured;* and so little difficulty has been experienced in its administration, that the proportion of cases in which it has decidedly disagreed has not amounted to four per cent.

The inoffensiveness of the oil implies the use of no process by which it can be deprived of its proper qualities. All that is required is, to obtain it *pure and fresh,* as it existed in the hepatic cells of the healthy fish when alive, without contamination by any process of putrefaction, roasting, boiling, or the like. On the contrary, the disgusting smell and taste, and dark color of the impure oil, proceed from the putrefaction and heat to which the livers are subjected, for the purpose of obtaining from them the utmost quantity of oil; hence it becomes highly rancid, and holds in solution or suspension various putrid and coloring matters derived from the corrupting cell and tissues of the liver.

It is not my intention to describe the details of the process by which the oil may be obtained in the greatest purity; but I may mention the following particulars, to which it is necessary to attend, in order to obtain a good product. The livers should be used as soon as possible after the death of the fish, every hour deteriorating the quality of the oil. The pale, plump livers should be preferred; those which are flabby and dark in color should be rejected as unhealthy. The livers, after being quickly pounded into a pulp, should be mixed with water of the temperature of about 120°, then filtered; and, after standing long enough, the oil is to be decanted from the filtered liquor, cooled to the temperature of 50°, and again filtered. The whole proces is to be accomplished with as little delay as possible, and in closed vessels, to prevent the air from giving to the oil the slightest degree of rancidity. For the same reason the vessels, in which the oil is preserved, should be full, well corked, and kept in a cool place. I recommend the second filtration after cooling, to remove the more solid part of the oil, the stearin and margarin, which not only further clears the oil by its separation, but by leaving a prepondrance of elain, gives to it more of that perfectly liquid and penetrative quality which promotes absorption and diffusion through the fluids and tissues of the body. My usual mode of administering cod-liver oil, is in doses of a tea-spoonful, gradually increased, (if the stomach bear it) to a table-spoonful, floating on some pleasant-flavored liquid, such as diluted orange wine, or the Infus. Aurantii Comp., with a little Tinct. and Syr. Aurantii. The vehicle should be suited to the taste and stomach of the patient; and much of our success in exhibiting the medicine will depend on our being able to keep the palate and stomach at peace with the oil. In numerous instances I have found that the addition of a little diluted nitric acid to the vehicle will make it more grateful to the palate, as well as serviceable to the stomach; and we may often combine with it other medicines which are not disagreeable, and thus fulfil the indications of palliating symptoms by their means. The fittest time for taking the oil, is from one to two hours after the three first meals of the day.—At this time the chyme is beginning to pass from the stomach into the duodenum; and it would appear that the oil passes quickly with it, for given at this time it causes none of those unpleasant eructations which are apt to occur when it is taken either before or with food. There is nothing in the oil for the stomach to digest; and the less it is brought into contact with it, and the sooner it passes out of it, the better. When it mixes with bile and pancreatic juice in the duodenum, its division and absorption begin and proceed, as in the case of all fatty maters. Herein, too, we see a reason why the oil does not agree so well either with the palate or stomach, when mixed in an emulsion, or combined with liquor potassæ, as recommended by some practitioners.

In conclusion, I repeat, that further observations, and longer time, are requisite to determine with accuracy the extent to which this agent can control or remove tuberculous disease of the lung; but I would state it as the result of extensive experience, confirmed by a rational consideration of its mode of action, that the *pure fresh Oil from the Liver of the Cod, is more beneficial in the treatment of Pulmonary Consumption than any agent, medicinal, dietetic, or regiminal, that has yet been employed.*

